# Lemierre's Syndrome (LS) Complicated by Cranial Nerve XII Palsy

**DOI:** 10.7759/cureus.38181

**Published:** 2023-04-26

**Authors:** Solabomi O Ojeniyi, Francis Ibukun, Philip Kanemo

**Affiliations:** 1 Internal Medicine, MedStar Union Memorial Hospital, Baltimore, USA; 2 Internal Medicine, Rapides Regional Medical Center, Alexandria, USA

**Keywords:** thrombosis of the internal jugular vein, anti coagulation, antibiotic, cranial nerve palsy (cnp), lemierre syndrome

## Abstract

Lemierre's syndrome (LS) is a rare medical condition that involves an acute oropharyngeal infection leading to septic thrombophlebitis of the internal jugular vein with embolic spread to organs like the kidneys, lungs, and large joints. Only very little literature has reported central nervous system involvement with LS. This is a case of 34-year-old woman with right-sided neck pain, swallowing difficulties, and a sore throat of 3 days duration at the time of presentation. CT of the neck with contrast showed a ruptured right peritonsillar abscess and thrombus in the right internal jugular vein suspicious of thrombophlebitis. The patient was managed for LS with IV antibiotics and anticoagulation. However, her clinical course was complicated by cranial nerve XII palsy, which is an extremely rare manifestation of LS.

## Introduction

Lemierre's syndrome (LS) refers to septic thrombophlebitis of the internal jugular vein. It starts with an oropharyngeal infection, then inflammation of the wall of the internal jugular vein, which results in an infected thrombus within the lumen of the internal jugular vein; this eventually leads to septic emboli and bacteremia. This condition is also called post-anginal sepsis and necrobacillosis [1-2]. 

Lemierre's syndrome was common in the pre-antibiotic period. It was rarely reported in the 60s and 70s during the widespread of penicillin for throat infections. Several works of literature in the 80s called it the ‘‘forgotten disease.’ It remains a rare disease with an incidence of about one per million persons per year. Recent literature showed that the incidence of LS is rising [2].

 *Fusobacterium necrophorum* and strictly Gram-negative anerobes, usually isolated in healthy young adults, are the most common microorganisms found in LS [3]. Antibiotic treatment is between 3 and 6 weeks, including metronidazole and β-lactam antibiotics. Commonly reported complications of LS are due to the hematogenous spread of the infection; pulmonary complications are also common, with symptoms like dyspnea, pleurisy, hemoptysis, and septic emboli. Septic arthritis or osteomyelitis can also arise from metastatic infection. 

We discuss an interesting case of LS complicated by right hypoglossal nerve palsy. 

## Case presentation

 A 34-year-old woman with no past medical history presented with complaints of right-sided neck pain and swallowing difficulties for the past three days. She also reported sore throat. She described her symptoms as insidious in onset and progressively worsened, with no known relieving or aggravating factors. She also endorsed associated fever but denied recent sick contact or IV drug use. Vital signs at presentation were significant for tachycardia, with a heart rate of 115 beats per minute. Physical examination was significant for erythema, swelling, non-tense, warmth to touch, tenderness on palpation of the right side of her neck, and no palpable lymph nodes. Laboratory studies at presentation were substantial for leukocytosis with neutrophil predominance (Table 1). 

**Table 1:** Laboratory findings at presentation.

Blood test results (units)	Patient value	Reference range
White blood count (k/uL)	19.7	4.0-10.8
Hemoglobin (g/gL)	14.1	11-14.5
Platelet count (k/uL)	272	145-400
Neutrophil count (%)	91.6	43-75
Absolute neutrophil count (k/uL)	18.1	1.7-8.1

**Figure 1:**
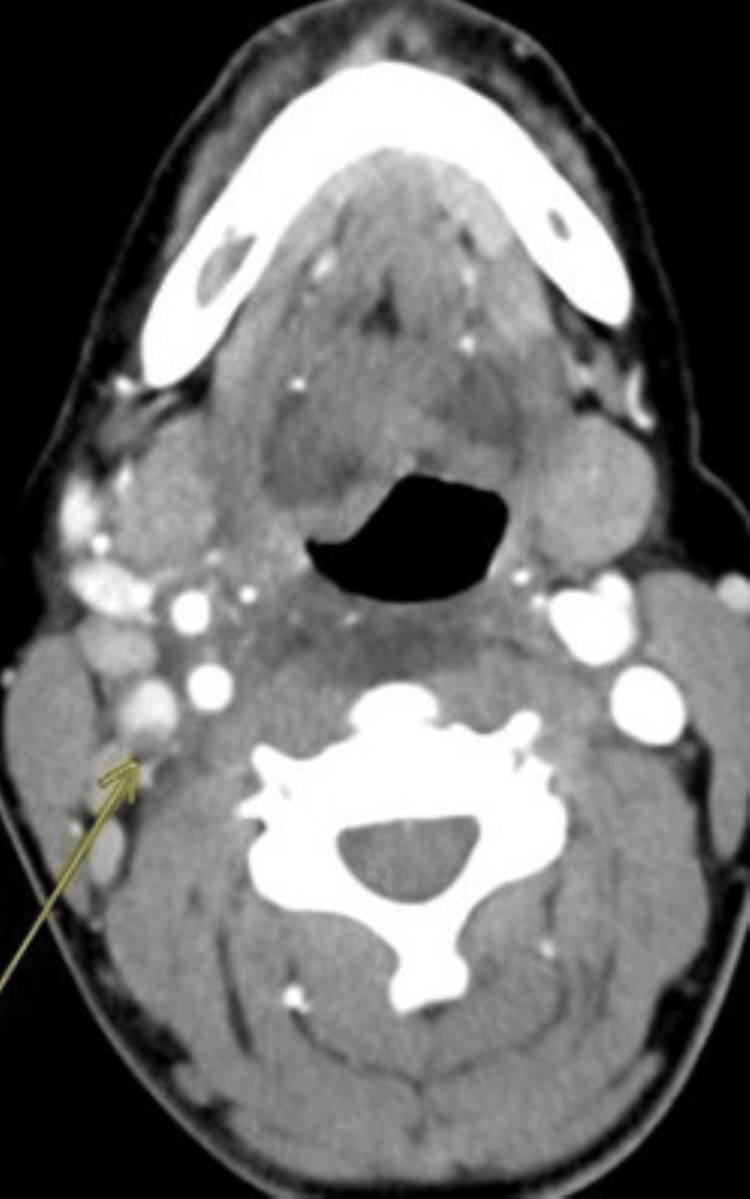
Axial view of CT with contrast demonstrating thrombus in the right IJV (yellow arrow). IJV, internal jugular vein

**Figure 2:**
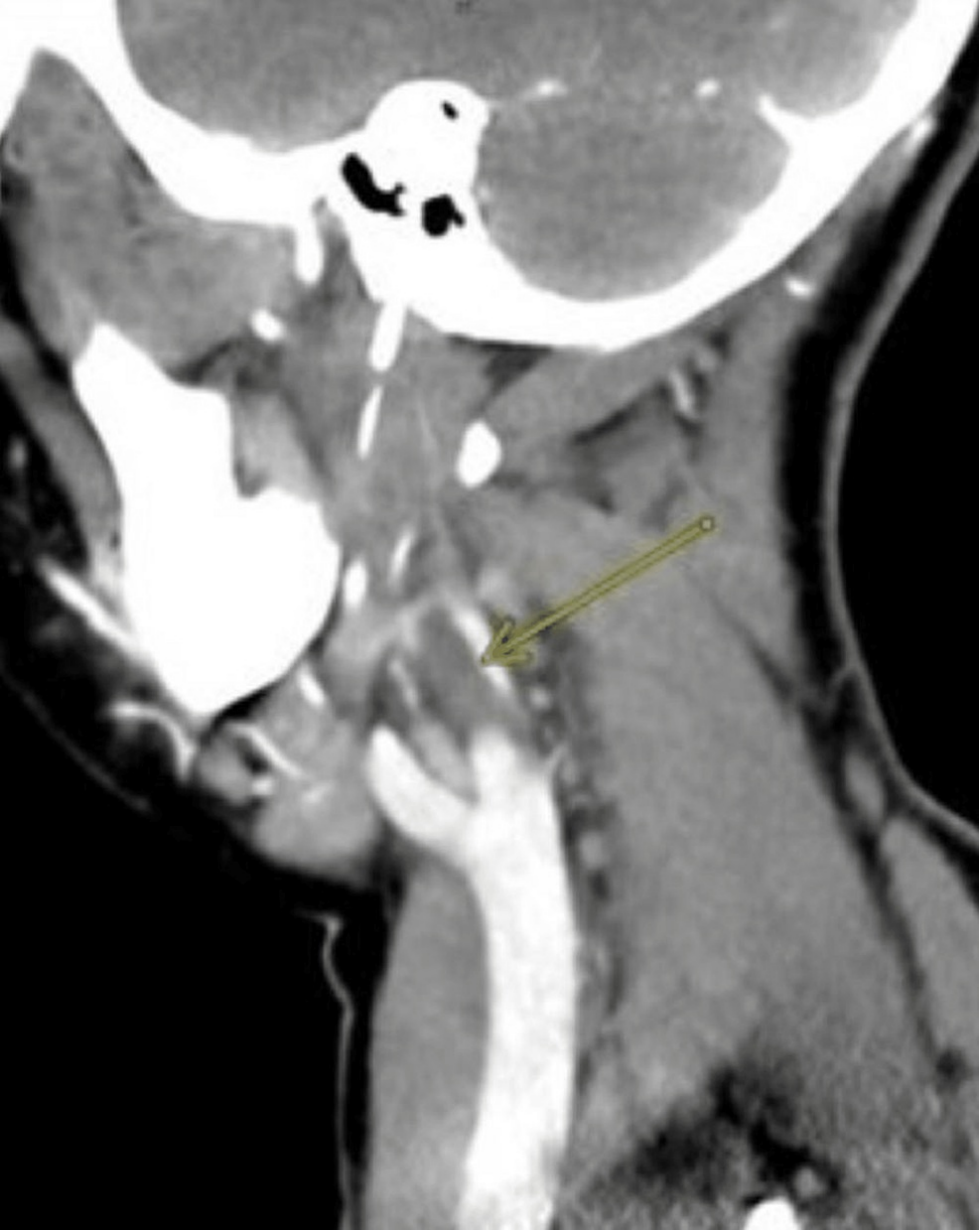
Sagittal view of CT with contrast demonstrating thrombus in the right IJV (yellow arrow). IJV, internal jugular vein

CT of the neck with contrast showed a recently ruptured right peritonsillar abscess with submucosal edema in the right oropharynx and hypopharynx in a prevertebral fluid collection and thrombus in the right IJV suspicious for thrombophlebitis (Figures 1-2).

**Figure 3:**
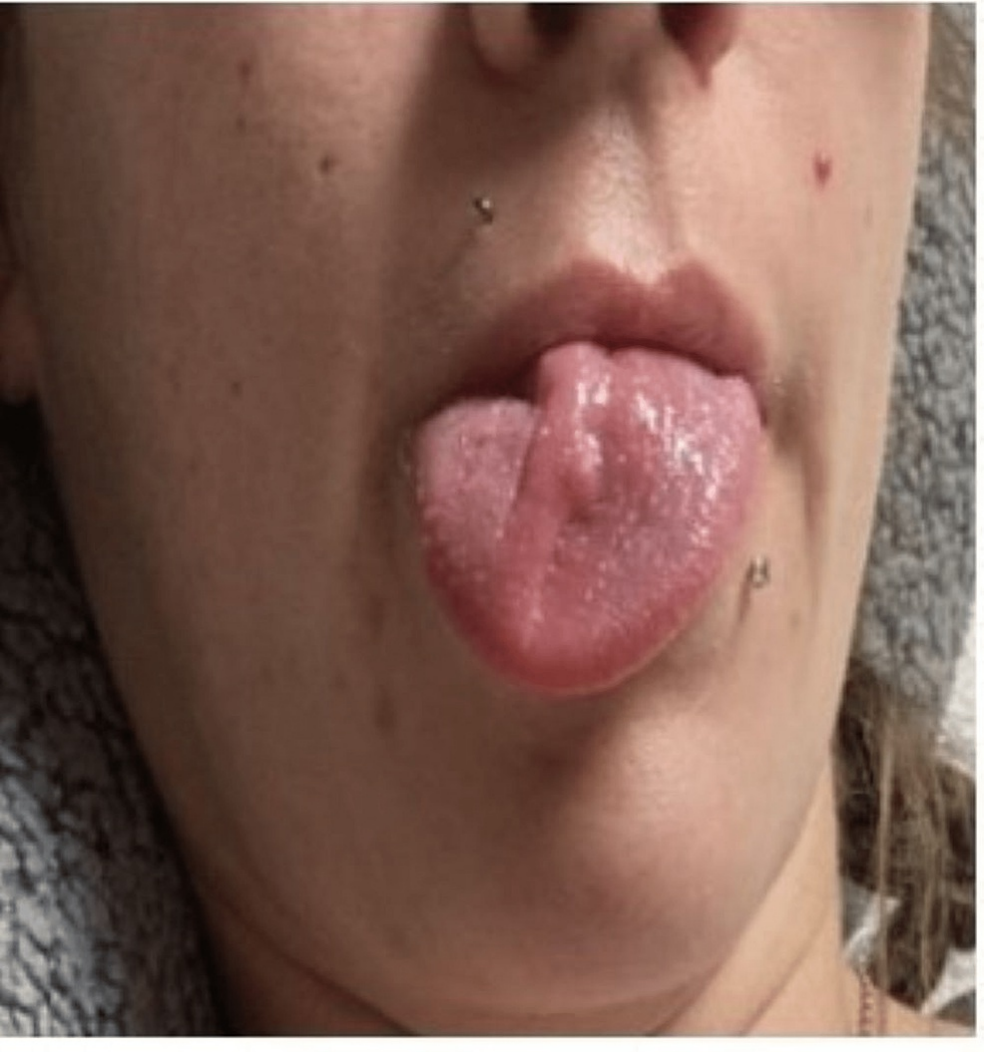
Right-sided tongue deviation 3 days after presentation.

She was diagnosed with LS based on her history and diagnostic findings from the CT scan. She was started on IV piperacillin-tazobactam 3.375 g every 6 h and initially on a heparin drip but transitioned to apixaban. Blood cultures grew no organism. However, on day 3 of admission, she complained of “speaking funny.” On examination, she was found to have right-sided tongue deviation (Figure 3) with weakness and atrophy on the right side of the tongue. MRI of the brain showed no intracranial abnormality. Magnetic resonance venography of the brain showed no central venous thrombosis.

Pathogenesis of cranial nerve XII palsy was likely secondary to direct effect from peritonsillar abscess. She was started on IV steroids as recommended by Neurology. A repeat ultrasound before discharge showed improvement in her right internal jugular (IJ) thrombus. She had received two weeks of IV antibiotics at discharge, and the right-sided tongue deviation was improving. She was eventually discharged home on oral metronidazole 500 mg twice daily and Omnicef 300 mg twice daily for an additional two weeks, a tapered dose of steroid, and Apixaban for three months with the plan to follow up with her Primary Care Physician, Neurology, and Vascular surgery. Unfortunately, she was lost to follow-up. 

## Discussion

Here we report a case of LS in an otherwise healthy young woman, diagnosed based on findings of the CT of the neck and neurologic complication of isolated right hypoglossal nerve palsy. 

Lemierre's syndrome (LS) has a low incidence rate of 1 case per 1 million people annually. The low incidence rate of LS results from an under-reported and under-recognized condition. It is common among the young age group, with about 90% of cases in individuals between 10 and 35 years of age [3]. Diagnostic criteria for LS include a history of anginal illness of the oropharynx within four weeks at the time of presentation, evidence of metastatic lesions in the lungs or other distant sites, and evidence of septic thrombophlebitis of internal jugular vein (IJV) or isolation of *F. necrophorum* from the blood or other sterile sites [3]. Although this index case had a negative blood culture, she met the criteria for LS based on CT findings of IJV thrombophlebitis and preceding anginal illness of the oropharynx. 

 Commonly reported complications of LS include metastatic spread like pleuropulmonary septic emboli, seen in 92% of cases with image findings of bilateral pulmonary infiltrates [4]. Pulmonary empyema, pleural effusion, or pulmonary abscesses develop in 17%-27% of patients [5-6]. Septic arthritis, mainly involving the hip, knee, and shoulder joints, is seen in 13%-27% of patients. Less frequent manifestations are osteomyelitis, muscular abscesses, skin and soft tissue abscess, and liver abscesses. Endocarditis and pericarditis are reported cardiac complications from LS but are rare [2]. Reported central nervous system complications include meningitis, cerebral infarcts or abscesses, and septic sinus venous thrombosis, which are rare and lethal. There are few reported cases of cranial nerve involvement in LS, but they are rare. Cranial nerve XII palsy is a rare complication of LS, with very few papers describing this. In this case, the etiology of the cranial nerve XII palsy could be from pressure or inflammatory effect, given CT neck findings of ruptured peritonsillar abscess and submucosal edema. 

The treatment principles for LS include appropriate antibiotic therapy and consideration regarding the need for surgical intervention and anticoagulation. Empiric treatment for LS should target F. necrophorum and oral streptococci. In addition, the regimen should contain an antibiotic resistant to beta-lactamase since *F. necrophorum* beta-lactamase production and treatment failure with penicillin have been reported [7-8]. 

 It is uncertain whether anticoagulation may reduce the propagation of thrombus or septic embolic events originating from IJV thrombosis; data are limited to case reports [9-10]. Thrombolytic therapy is an additional option when thrombosis progresses. There is minimal experience with this treatment in LS and no conclusive evidence of efficacy [9]. The need for interventional procedures like debridement of necrotic tissues and drainage of empyema should be guided by clinical circumstances. 

## Conclusions

Reports of cranial nerve XII palsy as a complication from LS are limited. Therefore, clinicians need to have a high index of suspicion diagnosing LS, and they also need to be aware of possible complications like cranial nerve XII palsy that may arise from LS. 
